# Intergenerational support and depressive symptoms among older adults in rural China: the moderating roles of age, living alone, and chronic diseases

**DOI:** 10.1186/s12877-021-02738-1

**Published:** 2022-01-27

**Authors:** Qian Sun, Youwei Wang, Nan Lu, Shiyan Lyu

**Affiliations:** 1 Department of Social Security, School of Public Administration, Hebei University of Economics and Business, Shijiazhuang, China; 2 Hebei Collaborative Innovation Center On Morality and Law-based Social Governance, Shijiazhuang, China; 3 Sau Po Centre on Ageing, The University of Hong Kong, Hong Kong, China; 4 Department of Social Work and Social Policy, School of Sociology and Population Studies, Renmin University of China, Beijing, China

**Keywords:** Intergenerational support, Depressive symptoms, Moderator, Older adults

## Abstract

**Background:**

While depressive symptoms are recognized as major mental health problems in later life, there is a lack of study in examining potential moderators in the association between intergenerational support and depressive symptoms, especially in social contexts with low socioeconomic status and inadequate formal public support. This study set out to examine the association between intergenerational support and depressive symptoms among older adults in rural Northeast China, and the potential moderating roles of age, living alone, and number of chronic diseases on this link.

**Methods:**

A quota sampling approach was used to recruit 448 respondents aged 60 and above from rural Chinese communities. Depressive symptoms were the dependent variable. Intergenerational emotional, instrumental, and financial support were the main independent variables. Age, living alone, and number of chronic diseases were the moderators. Multiple linear regression models with interaction terms were conducted to test the proposed model.

**Results:**

The results showed that intergenerational emotional support was significantly associated with depressive symptoms in older adults when instrumental and financial support and covariates were controlled (β = -0.196, p < .001). Age was found to have a significant moderating effect on the relationship between intergenerational instrumental support and depressive symptoms (β = -0.118, p < .05). Among older respondents aged 74.51 years and older, instrumental support was positively associated with depressive symptoms, but this association was not significant for younger respondents. Furthermore, living alone and number of chronic diseases suffered moderated the association between intergenerational financial support and depressive symptoms, which was statistically significant only for those living alone and with more chronic diseases (interaction term between living alone and intergenerational financial support: β = -0.082, p < .05; interaction term between number of chronic diseases and intergenerational financial support: β = -0.088, p < .05.

**Conclusions:**

The findings not only highlight the important role of intergenerational support in promoting mental health in later life in rural Chinese contexts, but also identify within-population heterogeneity in the identified associations. Policy and intervention implications are discussed.

## Background

The world population is ageing rapidly. In 2018, for the first time in history, adults aged 65 and older outnumbered children under five worldwide [1]. By 2050, one in six people globally will be over 65 (16%), up from one in 11 in 2019 (9%). The number of adults aged 80 years or older is also projected to triple, from 143 million in 2019 to 426 million in 2050. As the world’s most populous country, China has become one of the fastest aging nations. The number of people in China aged 65 and older reached 176.03 million in 2019, accounting for 12.6% of the total population [2].

Depressive symptoms are one of the major mental health problems among older people [3, 4]. They have been found to have an adverse impact on older adults’ physical health and mortality, and even to contribute to an increase in suicide rates [5]. For example, nearly half of all suicides in China take place among adults age 65 or above, and nearly 80% among rural residents [6]. Therefore, it is crucial to investigate the factors determining depressive symptoms in the older population in rural Chinese contexts.

The literature identifies a range of factors associated with depressive symptoms in later life, including but not limited to sociodemographic characteristics, educational attainment, income, living alone, adherence to traditional cultural values, cognitive function, and chronic disease [7–9]. We argue that intergenerational support is particularly important for older adults living in rural China, especially considering the dominant role of filial culture in these communities [10]. According to the filial piety value system, adult children, especially oldest sons, are expected to be obedient and take on the obligation of caring for their older parents when necessary [11, 12]. Older parents share these expectations. Given the two-tier nature of Chinese society, there is a large disparity between rural and urban areas in terms of social welfare benefits, health resources, social infrastructure, and education and employment opportunities [13]. This means that intergenerational support not only adheres to traditional filial culture, but also represents the primary source of care for older rural adults [14]. Only a limited number of studies have been conducted which simultaneously test the associations between multiple dimensions of intergenerational support and depressive symptoms, and the potential moderators of these associations. Therefore, the present study aimed to fill the research gap by examining the association between intergenerational support and depressive symptoms and the moderating role of age, living alone, and chronic disease in these relationships in a rural Chinese context.

### Intergenerational support and depressive symptoms

In this study, we conceptualize intergenerational support as a three-dimensional construct including instrumental, financial, and emotional dimensions [14–16]. Instrumental support denotes tangible forms of support, including personal care and undertaking household chores. Emotional support is an intangible construct referring to companionship and communication, which reflects intimacy and trust between parents and children. Financial support refers to the amount of financial transfer between generations of the family.

The findings of previous studies on intergenerational support and depressive symptoms are inconclusive. Some empirical studies have found that higher levels of intergenerational support can alleviate depressive symptoms [3, 17]. Lyons (2016) has demonstrated that instrumental and emotional support from family reduced psychological distress and alleviate the level of depressive symptoms [18]. Interventional studies have also suggested intergenerational programs could be used to promote mental health among older adults by making their lives more meaningful [19].

However, other studies have identified nonsignificant associations between intergenerational support and depressive symptoms/depression [20, 21]. Based on a sample of 3039 older adults living in urban areas of Beijing, Chen, and Silverstein (2000) found that the relationship between the provision of financial support and depressive symptoms among older adults was nonsignificant. Research has shown that these findings are largely a result of the various stressors that older adults confront in daily life, combined with the quality of interaction within intergenerational relationships [16, 21]. The findings suggest that several social and demographic factors could influence the relationship between intergenerational support and depression symptoms. Therefore, a more comprehensive analysis framework should be taken into consideration. Although there are abundant studies of the relationship between intergenerational support and depressive symptoms among older adults, few have yet considered how this association may vary across older adults in different age groups and those with different living arrangements and health status, especially in the developing country context. For example, cognitive function and physical health tend to decline with age, while levels of depressive symptoms tend to increase as people grow older and encounter a decline in their physical health [22]. From a needs-based perspective, intergenerational support from adult children could increase when their parents become older and develop more health problems [23]. Therefore, the relationship between intergenerational support and depressive symptoms could be different for different age groups (e.g., the young-old vs. old-old).

Living alone has been found to have detrimental effects on depressive symptoms in later life across countries and cultures. For example, older adults living alone tend to report more depressive symptoms than those living with others in the United States, South Korea, and Singapore [24–26]. Although the traditional multigenerational household living arrangement has undergone a great transition in modern Chinese society, living alone is not considered an ideal living arrangement for older adults [27]. For example, compared with those living with their adult children and grandchildren, older adults living alone might have fewer opportunities for social exchange with their offspring, and therefore receive less intergenerational support [28]. Therefore, intergenerational support could be particularly important in sustaining the physical and mental wellbeing of these older adults.

Furthermore, older adults with chronic diseases and illness burden report more depressive symptoms than those without chronic conditions [29–31]. As discussed above, older adults living in rural China tend to have less access to formal health resources, community services, and social welfare benefits compared to their urban counterparts [13]. In such circumstances, informal intergenerational support could play a more salient role in meeting older adults’ health needs, especially for those with multiple morbidities.

In summary, three gaps are identified in the research discussed above. First, although depression symptoms are one of the dominant risk factors for suicide and nearly 80% of suicides occur among rural residents in China, research exploring the variables determining depression symptoms in the older population in the rural Chinese context is limited. Secondly, few studies have examined associations between the multiple dimensions of intergenerational support and depressive symptoms in rural communities in China. This is important because intergenerational support has been found to be particularly important for the well-being of older adults in these areas, where the formal social support system is under-developed and there is an emphasis on filial piety. Thirdly, previous studies of intergenerational support and depressive symptoms have rarely considered the within-population heterogeneity of older age groups in the context of developing countries. Studying the potential moderating roles of age, living arrangements, and chronic disease on the association between intergenerational support and depressive symptoms in rural China will not only contribute to a more comprehensive understanding of healthy aging in a familial context, but also provide more information to help identify older adults at risk of depressive symptoms.

In order to fill these gaps, this study used data collected from a rural Chinese context to address the following research questions:

In order to fill these gaps, this study used data collected from a rural Chinese context to address the following research questions:

- Is receiving intergenerational support (i.e., instrumental, emotional, and financial support) from adult children associated with depressive symptoms in older parents living in rural Chinese communities? Based on this question, a research hypothesis was developed:

- H1: Receiving intergenerational support (i.e., instrumental, emotional, and financial support) from adult children is negatively associated with depression symptoms among older parents living in rural Chinese communities.

(b) Does age play a moderating role in the relationship between intergenerational support and depressive symptoms? Based on this question, a research hypothesis was developed:

- H2: Age strengthens the relationship between intergenerational support (i.e., instrumental, emotional, and financial support) and depression symptoms. Specifically, intergenerational support plays an important role in alleviating depression symptoms among older parents in rural Chinese communities, especially as they age.

(c) Does living alone play a moderating role in the relationship between intergenerational support and depressive symptoms? Based on this question, a research hypothesis was developed:

- H3: Living alone strengthens the relationship between intergenerational support (i.e., instrumental, emotional, and financial support) and depression symptoms. Specifically, intergenerational support plays an important role in relieving depression symptoms among older parents in rural Chinese communities, especially when they live alone.

(d) Does chronic disease play a moderating role in the relationship between intergenerational support and depressive symptoms? Based on this question, a research hypothesis was developed:

- H4: Chronic diseases strengthen the relationship between intergenerational support (i.e., instrumental, emotional, and financial support) and depression symptoms. Specifically, intergenerational support plays an important role in alleviating depression symptoms among older parents in rural Chinese communities, especially as the number of chronic diseases affecting them increases.

## Methods

### Sampling

 The data for this study were derived from a community survey jointly administered by (Blinded for review) in 2019. A quota sampling method was used to select a sample from Dongliao county, Jilin province, China. Dongliao county is the rural area of Liaoyuan city in northeast China. There are around 335,700 residents, with an average disposable income of $2209 per person per year in 2018. People aged 60 years or older account for 21.33% of the population (national average 17%), and the majority of older residents have lived in local rural communities for decades. Firstly, we randomly selected 16 of the 235 administrative villages in Dongliao county. Secondly, based on referrals from the village committee, 30 respondents aged 60 or above were selected in each village. The age and gender ratios of the respondents were controlled according to the statistics in the sixth national census. The respondents needed to meet the following criteria; (1) have local household registration status; (2) be aged 60 or older; and (3) have lived in a local village for more than 180 days in the past 12 months.

 Trained interviewers conducted face-to-face interviews at the respondents’ homes and local community centers. All respondents signed an informed consent form before starting the survey. They were also informed of their right to withdraw from the study at any time. A total of 486 people were interviewed, of whom 482 completed the exercise. The Short Portable Mental Status Questionnaire (SPMSQ) was also used to assess the respondents’ cognitive function [32]. After excluding the respondents who did not pass the cognitive test (n = 24) and those who did not have adult children (n = 10), the sample size used in the analysis was 448. Ethics approval was obtained from the Ethics Committee of University of Hong Kong (Reference No.: EA: 2,003,026).

### Measurement

#### Outcome variable

The 10-item Center for Epidemiologic Studies Depression Scale (CES-D-10) was used to measure the levels of depressive symptoms among older respondents in the past week at the time of survey [33]. This scale includes negative mood items such as “I am troubled by some small things,” and “I feel lonely;” positive items such as “I am happy,” and “I am hopeful for the future;” and somatic syndromes such as “My sleep is not good.” Answers are collected using a five-point scale (0 = rarely or none of the time, 1 = not too much, 2 = almost a half, 3= most of the time, 4 = almost every day). The summed score (ranging from 0 to 40) represents the level of depressive symptoms, with a higher score indicating a higher level of symptoms. The Cronbach’s alpha estimate for the CES-D-10 is 0.845 for this sample.

#### Intergenerational support variables

Given the majority of the respondents had more than one adult child (range = 1-9), the respondents were asked about their relationships and interactions with each of them. In this study, intergenerational support is assessed along three dimensions; instrumental, emotional, and financial support [14, 16]. For each child, instrumental support was measured by the following item: “How much instrumental support has your child has given you in the past 12 months?” The responses were collected using a five-point Likert scale ranging from 1 = almost never to 5 = almost every day. Financial support was assessed by the item “How much financial support did you receive from your child in the past year?” Emotional support was measured using three items based on the Intergenerational Solidarity Inventory [34], namely; (a) “Did you feel emotionally close to your child?”; (b) “Is this child willing to communicate with you in terms of your worries and troubles?”; and (c) “Did you get along with this child”. Answers to each item were assessed using a five-point Likert scale, ranging from 1 = complete stranger/refused to listen/very poor, to 5 = very close/every time/excellent. The summed score represents the level of emotional support (range = 3-15; Cronbach’s alpha = 0.918).

 Given that adult children in rural China often share the obligations of care for their older parents, we used the summed scores of instrumental/financial support for all the respondent’s adult children to represent the levels of instrumental/financial support received. Furthermore, filial piety culture places great emphasis on adult children showing respect and obedience to their parents. Therefore, we used the average score of emotional support from all adult children to represent the level of such support between generations.

#### Moderator and covariates

Age was measured in years. The respondents were asked whether they lived alone or with others at the time of survey (0 = living with others; 1 = living alone). The respondents were also asked whether they had any medically diagnosed chronic diseases, based on a list of 14 chronic diseases that are common in later life (e.g., hypertension, heart diseases, and arthritis; 0 = no; 1 = yes). The responses were summed to represent the number of chronic diseases suffered by the respondent.

Gender, marital status, and educational attainment were recoded as binary variables (0 = male,1 = female; 0 = other marital status, 1 = married; 0 = no formal education, 1 = primary school education and above). Income was measured by a single question: “How much is the annual household income of you and your spouse in the past year?” The respondents were asked to report the number of their children. The 10-item Barthel Index was used to measure activities of daily living (ADL [35]). The responses to this index are assessed using a three-point scale (0 = very difficult; 5 = quite difficult, assistance needed; 10 = no difficulty at all). Summed scores were calculated to represent the respondents’ level of ability to complete ADL. The higher the score, the greater their ability to live independently. The Cronbach’s alpha estimate for the scale in this study is 0.837. Finally, cognitive function was assessed using the 10-item Short Portable Mental Status Questionnaire (SPMSQ: range = 6-10). The cutoff point of this scale is 7 for those who completed a college education and 6 for those who completed high school or lower.

### Data analysis

Descriptive statistics were computed for the sociodemographic characteristics of the sample and factors related to depressive symptoms. Multiple linear regression analysis was then used to test whether age, living alone, and number of chronic diseases moderated the association between intergenerational support and depressive symptoms.

Collinearity, normality, autocorrelation, and outliers were checked before processing the multiple linear regression analysis. The values of the variance inflation factor (VIF) (VIF values above 10 would indicate multicollinearity) of independent variables in the present regression model ranged from 0.00 to 2.11, indicating no multicollinearity [36]. Homoscedasticity and normality assumptions of the multiple linear regression model was tested based on residual plots. It showed that the scatter of the standardized residuals is uniformly distributed around the top and bottom sides of the line of the standardized residual ei=0 in a certain region, and will not vary with the change of the standardized predicted value. White Heteroskedasticity Test was also conducted by Stata 16.0. The results showed the p value is 0.3243, larger than 0.05. Therefore, the homoscedasticity and normality was satisfied [37]. The Durbin-Watson value of the present linear regression model is 1.80, indicating there is no autocorrelation [38]. Outliers were screened through multivariate outlier analysis; the Mahalanobis distance results revealed that no cases had significant D^2^. None of the variables were missing more than 2.5% of the overall number of values, and those that were missing followed a random pattern. According to the criteria proposed by Tabachnick and Fidell [39], less than 5% missing data is considered acceptable. Missing values in this dataset were handled in the listwise deletion through the linear regression process.

Depressive symptoms were regressed on the covariates in the first model. Intergenerational support variables were entered in the second model after covariates were controlled. In the third model, we entered nine interaction terms of three moderators and three independent variables [40]. Interaction terms with nonsignificant coefficients were deleted from the model using the backward elimination method. The final regression model included one depressive symptom variable, three intergenerational support indicators, 10 control variables, and three interaction terms. All interaction terms were mean-centered to reduce multicollinearity, The analyses were carried out using IBM SPSS Statistics 25.

The Johnson-Neyman technique was employed to identify regions where the effect of intergenerational support on depressive symptoms became or ceased to be statistically significant across the range of the moderating variable [41]. The Johnson-Neyman technique was regarded as a more advanced approach to visualize and report the moderation effect when the moderator is continuous [42]. It has the potential to provide the researcher with additional information about the region of significance with different treatment effects. A slight generalization can express, as a function of M, the lower and upper bounds for confidence bands estimating the effect of X on Y [43]. A significance level of 0.05 was used in the current study.

## Results

### Sample characteristics

The descriptive characteristics of respondents are presented in Table 1. Around half of the sample was male, 71.2% were married, 81.0% lived with others, and 36.4% had not received any formal education. The average age of respondents was 69.5 years. On average, each had 2.5 adult children. In terms of income, 66.3% of respondents had an annual household income of lower than 1,5000 RMB. Around 14.1% had at least one ADL limitation, and 24.1% did not have any chronic diseases at the time of survey. Furthermore, 14.8% and 24.1% of the respondents had not received any instrumental or financial support from their adult children in the past 12 months, respectively. Around half (48%) considered their relationships with their adult children as very close or excellent. Finally, around one-fifth of the respondents did not report any depressive symptoms.

**Table 1:** Characteristics of the study sample (n=448)

Variable	*n* (%)	*Mean* (SD)
Age		69.5 (6.3)
60-64 years	98 (21.9)	
65-69 years	164 (36.6)	
70-74 years	101 (22.5)	
75 and older	85 (19.0)	
Gender		
Male	226 (50.4)	
Female	222 (49.6)	
Marital status		
Married	319 (71.2)	
Other status	129 (28.8)	
Education		
Illiterate or no formal education	163 (36.4)	
Primary school education and above	285 (63.6)	
Annual household income		15627.0(16640.0)
Lived with others	363 (81.0)	
Number of children		2.5(1.3)
ADLs		98.1 (6.7)
Cognitive function		9.1 (1.1)
Number of chronic diseases		1.7 (1.7)
Instrumental support		7.9(5.5)
Emotional support		13.7(1.6)
Financial support		3523.4(6052.5)
Depressive symptoms		5.6 (5.4)

Notes: ADLs = Activities of Daily Living; n = number; SD = standard deviation

### Multiple linear regression models

Table 2 presents the results of the multiple linear regression analyses. All variance inflation factor (VIF) estimates were lower than 4, and tolerance estimates were higher than 0.2 [44]. Therefore, multicollinearity is not a problem in this analytic model. In the first model, gender, living alone, income, ADL, cognitive function, and number of chronic diseases were found to be significantly associated with depressive symptoms (gender: b = 1.408, SE = 0.496, β = 0.127, p < .01; living alone: b = 2.695, SE = 0.773, β = 0.176, p < .01; income: b = -0.998, SE = 0.262, β = -0.171, p < .001; ADL: b = -0.115, SE = 0.036, β = -0.142, p < .01; cognitive function: b = -0.843, SE = 0.225, β = -0.168, p < .001;number of chronic disease: b = 0.808, SE = 0.144, β = 0.247, p < .001). Three indicators of intergenerational support were added in the second model. After controlling for covariates, intergenerational emotional support was significantly associated with depressive symptoms (b = -0.668, SE = 0.157, β = -0.196, p < .001). The associations between intergenerational instrumental support, financial support, and depressive symptoms were statistically nonsignificant (p > .05).

**Table 2:** Multiple Regression for Intergenerational Support and Depressive symptoms

Variable	Model 1				Model 2			Model 3		
	B	SE	β		B	SE	β	B	SE	β
(Constant)	33.109	4.566			40.832	4.937		41.040	4.864	
Age	-0.042	0.049	-0.048		-0.067	0.049	-0.076	-0.065	0.048	-0.074
Gender	1.408	0.496	0.127**		1.460	0.486	0.132**	1.507	0.480	0.136**
Marital status	0.052	0.642	0.004		-0.130	0.634	-0.011	-0.149	0.625	-0.012
LA	2.695	0.773	0.176**		2.249	0.766	0.146**	2.169	0.755	0.141**
Education	0.238	0.514	0.021		0.297	0.505	0.026	0.305	0.498	0.027
Income	-0.998	0.262	-0.171***		-0.765	0.266	-0.131**	-0.786	0.262	-0.135**
ADLs	-0.115	0.036	-0.142**		-0.118	0.036	-0.146**	-0.118	0.035	-0.146**
Cognition	-0.843	0.225	-0.168***		-0.776	0.221	-0.154**	-0.741	0.219	-0.147**
NOD	0.808	0.144	0.247***		0.801	0.142	0.245***	0.796	0.140	0.243***
NOC	-0.082	0.233	-0.019		-0.451	0.323	-0.104	-0.594	0.320	-0.138
IES					-0.668	0.157	-0.196***	-0.685	0.155	-0.201***
IIS					0.128	0.071	0.127	0.084	0.073	0.083
IFS					-0.102	0.068	-0.065	-0.084	0.067	-0.053
Age*IIS								0.012	0.005	0.118*
LA*IFS								-0.359	0.177	-0.082*
NOD * IFS								-0.079	0.036	-0.088*
*F*	16.284***			14.790***				13.358***		
Adjusted *R*^*2*^	0.264			0.297				0.318		

Notes: *p < .05. **p < .01. ***p < .001; B=unstandardized regression coefficient; β = standardized regression coefficient; LA = living alone; NOC = Number of children; NOD = number of chronic diseases; IES = Intergenerational emotional support; IIS = Intergenerational instrumental support; IFS = Intergenerational financial support

In the third model, three significant interaction terms were identified (between age and intergenerational instrumental support: b = 0.012, SE = 0.005, β = -0.118, p < .05; between living arrangement and intergenerational financial support: b = -0.359, SE = 0.177, β = -0.082, p < .05; between number of chronic diseases and intergenerational financial support: b = -0.079, SE = 0.036, β = -0.088, p < .05). All the other interaction terms, between age, living arrangement, health and intergenerational support indicators were statistically nonsignificant, (p > .05).

**Fig. 1:**
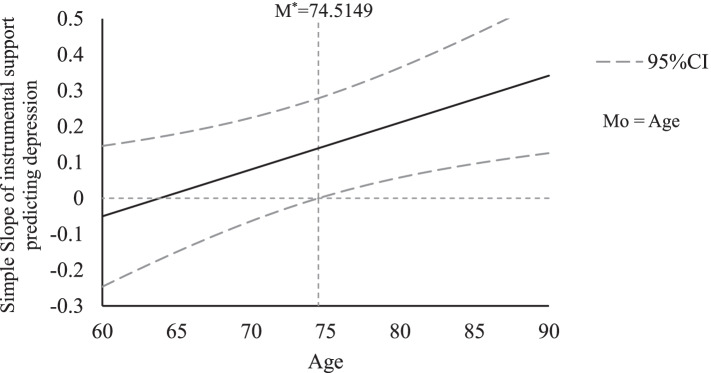
At a 95% confidence level, the effect of instrumental support on depressive symptoms is significant when Mo≥M^∗^,where M^∗^=74.5149. Notes: Mo=Moderator variable; M*=Critical value of moderator variable

**Fig. 2:**
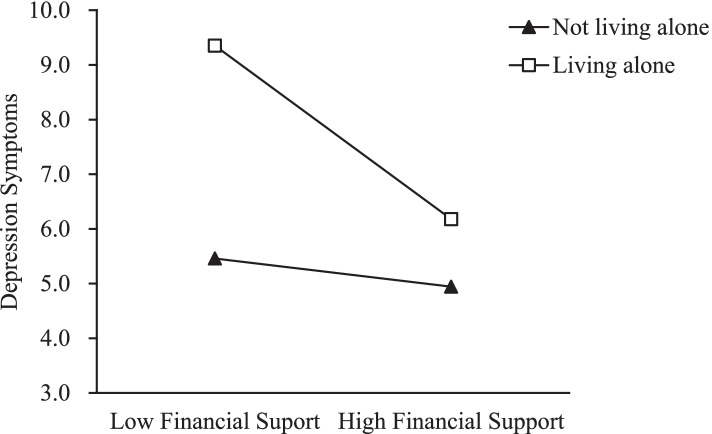
Simple slope diagram of the moderator effect of living alone on the association between intergenerational financial support and depressive symptoms

**Fig. 3:**
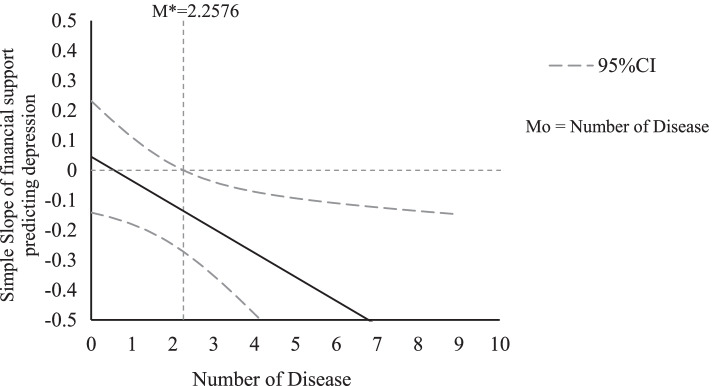
At a 95% confidence level, the effect of financial support on depressive symptoms is significant when Mo≥ M^∗^,where M^∗^=2.2576. Notes: Mo=Moderator variable; M*=Critical value of moderator variable

A Johnson-Neyman (JN) technique and plot were conducted to further examine the moderating effects. The JN technique allows us to determine the values of a moderator for which the effect of the independent on the dependent variable becomes or ceases to be significant [45]. The JN graph shows the values of the moderator when the effect of intergenerational support on depressive symptoms becomes significant [43]. Figures 1, 2 and 3 present the JN plots for the three variables (age, living arrangement, and number of chronic diseases) having a significant moderating effect on the association between intergenerational support and depressive symptoms.

As shown in Table 2, age and intergenerational instrumental support were not significantly associated with depressive symptoms. However, the interaction term between the first two variables did show a significant association with the latter. Figure 1 shows that the link between intergenerational instrumental support and depressive symptoms was only statistically significant among respondents aged 74.51 years and above. For this older age group, higher levels of instrumental support were associated with higher levels of depressive symptoms. This association was statistically nonsignificant in the younger group (i.e., those below 74.51 years).

Living alone was positively associated with depressive symptoms. Intergenerational financial support was not significantly associated with depressive symptoms in the second or third models. However, the interaction term was negatively associated with depressive symptoms. According to Fig. 2, this association was significant for older adults who lived alone, but not those who lived with others.

As shown in Table 2, the number of diseases suffered was significantly associated with depressive symptoms. Intergenerational financial support was not a significant predictor of depressive symptoms in the second or third models. The interaction term between number of chronic diseases and depressive symptoms, however, was statistically significant. In Fig. 3, it can be seen that that the association between intergenerational financial support and depressive symptoms was significant among older respondents who had 2.26 or more chronic diseases. For those with fewer, it was nonsignificant.

## Discussion

This study is one of the first attempts to examine the moderation effects of age, living alone, and number of chronic diseases in the associations between intergenerational support and depressive symptoms for older adults living in rural China. The findings provide new evidence for family-based policies and interventions to promote mental health in later life, especially among older rural-dwelling adults, who are socially and economically disadvantaged compared with their urban counterparts.

Consistent with the findings of previous studies, the present study has shown that intergenerational support, especially the emotional support dimension, could lead to a reduction in levels of depressive symptoms. Intergenerational emotional support not only represents a sense of belonging, concern and support, but also adheres to the traditional filial cultural expectations (i.e., obedience and respect). These factors could further reduce depressive symptoms among older adults [46–48].

While previous studies have shown mixed findings in terms of the association between intergenerational instrumental and financial support and depressive symptoms [3, 16, 17, 20, 21], this study makes a new contribution to the literature by testing the moderation effects of age, living alone, and number of chronic diseases on these associations.

Firstly, an interesting finding is that the interaction term age and instrumental support is significantly and positively associated with depressive symptoms. To be more specific, instrumental support was not associated with depressive symptoms among adults younger than 74.51 years, but it was positively associated with such symptoms among those aged 74.51 years and older. This finding indicates that older adults in rural areas tend to be more psychologically responsive to instrumental support from their adult children than those under 74.51 years of age. It also lends support to the strong familistic and filial piety values of traditional Chinese culture, especially for the oldest individuals. These values set an expectation that older individuals will live at home for as long as possible until the end of their lives [49], and will be cared for by their offspring rather than by other kin, domestic helpers, or in institutions. Our results suggest that instrumental support behaviors by their children, such as doing the housework, helping with cooking, and fixing appliances, may be particularly important for older parents’ sense of being cared for, being the object of concern, and being respected. These findings could therefore suggest that 74.51 is a critical age above which the older rural adults among our respondents needed their adult children to provide additional instrumental support to meet their daily care and psychological needs.

Secondly, although the relationship between intergenerational financial support and depressive symptoms was nonsignificant in the second model, the relationship varied according to living arrangement and chronic disease status. Living alone runs contrary to the traditional multigenerational household arrangements for older adults. It is not only associated with lower levels of social exchange and support with adult children, but also does not fit with the traditional filial culture and filial expectations held by older adults. Therefore, our finding emphasized the importance of financial support in cases where the older parents and their adult children had limited opportunities for face-to-face interaction and could not easily facilitate other patterns of intergenerational support (i.e., emotional and instrumental support). Providing financial support to those living alone perhaps helps to create a sense of self-worth and to avoid feelings of abandonment among older individuals in rural China, and this benefits their psychological wellbeing. It is reasonable to assume that older adults with a higher incidence of chronic diseases (n > 2.26) might confront more financial stress compared to their healthy counterparts. Under such circumstances, financial support from adult children not only represents a valuable supportive resource to meet the older person’s daily and health care needs, but also meets their filial expectations to some extent. Financial support from adult children may create economic security for older adults in poor health and relieve any psychological stress related to financial hardship. Moreover, such support may be particularly important for rural residents whose health literacy is inadequate, therefore making a greater contribution to their ability to access treatment for chronic conditions. The provision of financial support could be a means by which adult children can demonstrate they care for their sick parents, enhancing the parents’ sense of being important to their children.

The findings also have policy and intervention implications. Firstly, depressive symptoms seem to be a prevalent mental health problem among older, rural Chinese residents and intergenerational support is an important protective factor. This implies that policy makers and community service designers could expand the target population when addressing the mental health needs of older adults. On one hand the following criteria might be used to identify rural older populations at risk of depressive symptoms and depression: families with poor intergenerational relationships, with parents aged 74.51 and older, who are living alone, and have 2.26 or more chronic diseases. On the other hand, service planners could establish case management and a range of mental health improvement strategies with family-directed approaches for older adults in rural areas. Secondly, the provision of instrumental support from adult children could be an effective strategy to mitigate depressive symptoms among older rural Chinese adults aged 74 and above. Community social workers and other professionals could promote mental health among older adults by conducting interventions to nurture family obligations and social exchanges across the generations. For social services and volunteer services, which act as alternative sources of instrumental support, involving adult children in the decision-making and service delivery process is also encouraged. Thirdly, older adults living alone and with relatively high levels of chronic conditions require more financial support from their adult children, and this helps to relieve depression symptoms. Community social workers could highlight this need and facilitate financial support from adult children to the older individuals. Policy-makers and social service providers implementing proactive employment policies may need to give priority to people with older parents in need of support. Moreover, those among the working population who have parents in poor health could perhaps be included as a qualification for individual income tax return in China.

### Limitations

There are several limitations of this study. Firstly, the data is cross-sectional in nature and so the causal relationship between intergenerational support and depressive symptoms cannot be examined. In future studies, a longitudinal approach will be needed to address this issue. Secondly, a quota rather than random sampling approach was used to recruit the respondents in this study. This limits the power to generalize the findings. In particular, the data were collected from individuals in Dongliao county, Jilin province, and so may not be capable of being generalized to China’s rural older population as a whole. Third, the measurement of financial support only assessed income. Future studies should use other instruments to measure related variables such as asset and expenditure. Lastly, given the relatively small sample size, we did not recode living arrangement as a categorical variable with multiple categories (e.g., living alone, living with spouse only, living with grandchildren only, living with both spouse and adult children). Future research with larger sample sizes is needed to examine the moderating effects of living arrangements on the relationship between intergenerational support and depressive symptoms in later life across different social and cultural contexts.

## Conclusions

Intergenerational emotional support was found to have a stronger influence on depressive symptoms in older adults than instrumental and financial support. Furthermore, the associations between intergenerational instrumental and financial support and depressive symptoms in later life were found to vary by age, whether or not the older adult lived alone, and the number of chronic diseases suffered. While instrumental support was only associated with increased depressive symptom levels among adults aged 74 years and above, and financial support was associated with depressive symptoms among older adults with relatively more chronic diseases and those living alone only. Community-based services should be developed not only to enhance emotional bonding and exchanges across generations, but also to provide individualized support so as to meet the social needs of older adults with different demographic characteristics, living arrangements, and health status.

## Data Availability

The datasets used and/or analysed during the current study are available from the corresponding author on reasonable request.

## Abbreviations

SPMSQ: Short Portable Mental Status Questionnaire
JN: Johnson-Neyman
VIF: variance inflation factor
ADLs: Activities of Daily Living
SD: Standard deviation
LA: Living alone
NOC: Number of children
NOD: Number of chronic diseases
IES: Intergenerational emotional support
IIS: Intergenerational instrumental support
IFS: Intergenerational financial support
